# Predictors of insecticidal net use among internally displaced persons aged 6-59 months in Abuja, Nigeria

**DOI:** 10.11604/pamj.2018.29.136.13322

**Published:** 2018-02-27

**Authors:** Joan Ejembi, Olufemi Ajumobi, Muhammed Sani Ibrahim, Saad Ahmed, Adebola Tolulope Olayinka

**Affiliations:** 1 Ahmadu Bello University, Zaria, Kaduna State, Nigeria; 2 Nigeria Field Epidemiology and Laboratory Training Programme, Abuja, Nigeria; 3 African Field Epidemiology Network, Abuja, Nigeria

**Keywords:** Internally displaced persons, long-lasting insecticidal nets, Child, Nigeria

## Abstract

**Introduction:**

Malaria is the commonest cause of morbidity and mortality among displaced populations especially children in endemic countries. Nigeria, an endemic country, has had increase in internally displaced persons (IDP) due to insurgency. utilization of long-lasting insecticidal nets (LLIN) is a key strategy employed to achieve global targets of malaria elimination and its effectiveness is determined by utilization, coverage and maintenance. We determined the coverage and utilization of LLIN among children aged 6-59 months at IDP camps and its predictors.

**Methods:**

A cross-sectional study was conducted among children aged 6-59 months at the three IDP camps in Abuja. We collected data on socio-demographic characteristics, LLIN ownership and utilization using computer-aided interview, complemented by direct observation of nets. We defined universal LLIN coverage as the proportion of households with one LLIN to two persons and utilization as an eligible child sleeping under the LLIN the night preceeding the survey. Bivariate analysis was done at p < 0.25 and logistic regression at 5% level of significance.

**Results:**

Overall, 393 children were enrolled with mean age of 33.3 ± 17.4 months, 51.6% were female. Household LLIN ownership was 76.7.5%, universal coverage 11.2% and utilization 89.7%. Independent predictors of LLIN utilization were LLIN hung at sleeping area (adjusted OR: 99.9, CI: 22.7 – 438.8) and type of camp site (adjusted OR: 8.2, CI: 2.5 – 27.4).

**Conclusion:**

LLIN utilization was high in these IDP camps despite low coverage. LLIN distribution and hanging campaigns are recommended to reduce malaria transmission in the IDP camps.

## Introduction

Globally, in 2015, Malaria accounted for an estimated 214 million cases and 438 thousand deaths [1]. Nigeria and the Democratic Republic of Congo (DRC) accounted for more than one third of the malaria attributable deaths globally [1,2]. Both countries are not only endemic for malaria, but also suffer from conflict and violence resulting in displacement of thousands of people [3,4]. It has been shown that malaria strives during conflict and disaster in malaria endemic countries [2,3,5].`This is partly due to the breakdown of health services and interruption of control programs. In addition, people displaced from regions of lower to higher endemicity are prone to more severe disease while those displaced from regions of higher endemicity can enhance disease transmission [2,3]. Internally displaced persons are a disadvantaged population, prone to challenges such as insecurity, limited access to health care, infectious and epidemic prone diseases. Since 2000, there has been an upsurge of internally displaced persons (IDPs) in Nigeria due to insurgency in the North East, communal clashes in the North Central and South West and attacks from Fulani herdsmen [4] and this contribute to the high number of IDPs in Nigeria. Furthermore, Nigeria has been categorised among countries which may suffer from protracted conflict and strife [3].

Malaria has been identified as a common cause of morbidity and mortality among displaced populations especially women and children [6,7]. This may worsen the already poor under-five indices which was reported as 128 deaths per 1000 live births in 2015, one of the worst in the world [8]. Malaria is a major contributor to under-five morbidity and mortality in un-displaced and even more so in displaced populations [2,9]. However, most IDP camp evaluations focus on shelter, "Water, Sanitation , Hygiene" (WASH) and malnutrition [5,10,11]. Only few countries include IDPs in their national malaria control programme plan, even in malaria endemic countries [10,12]. The Global Technical Strategy aims to eliminate malaria from at least 35 countries in which it was transmitted in 2015 [13]. Four main preventive strategies have been instituted for its control amongst which the use of long-lasting insecticidal nets (LLIN) is considered the corner stone with a preventive effect comparable to vaccination [2,14–16]. Effectiveness of LLIN depends on the coverage, utilization, maintenance and timely replacement of nets among other factors [2,13]. Preventive measures utilised among IDPs and refugees include LLIN, insecticide treated tarpaulins, mosquito proofing of night shelters and insecticide treated net wall hangings [17,18]. Of these, LLINs are available in the country and distribution campaigns in the general populace has been done since 2010. This study was conducted to determine the coverage of LLIN and predictors of LLIN utilization among internally displaced children aged 6-59 months living at IDP camps in Abuja, Nigeria.

## Methods

### Study area and design

The study was conducted in Abuja, the nation’s capital. It has an altitude of 477mm, average temperature and rainfall of 25.7°C and 1389mm respectively. The climate is tropical with two main seasons. The rainy season from May to October and dry season from November - February, Malaria transmission occurs all year round with the peak transmission from July - September. The camps at New Kuchingoro and Durumi Area One are camp - like sites in open air settlements with tents made of tarpaulin in fields and farm lands in rural areas, lacking basic amenities while the Wassa camp is a settlement site where the displaced persons are housed in abandoned buildings. No health facilty is available at any of the camps, though the camps at Wassa and Durumi Area one have a dispensary. The only services provided are consultation for common ailments and drug dispensing. Displaced persons mostly access health care from facilities outside the camps. Drugs at the dispensary are usually donated by non-governmental organisations (NGOs) and philanthropists however the supply is irregular. A cross-sectional study was conducted among children aged 6-59 months at the three IDP camps in Abuja from July – October 2016.

### Study population

All children aged 6-59 months living within Abuja IDP camps excluding those unavailable at the time of survey.

### Data collection

Two structured interviewer-administered questionnaires (household and child) adapted from the 2015 Nigeria Malaria Indicator Survey were used to collect data on both the household and child [19]. Data were collected using Open Data Kit (ODK) on android-based phones. Trained interviewers administered the questionnaires to collect information on the socio-demographic characteristics of the caregivers, number of household members, state of residence before displacement, reason for displacement, duration of stay in camp, LLIN ownership and type of housing or shelter structure. Also data on demographic characteristics, and utilization of LLIN was collected with the child questionnaire and direct observation of nets to determine if LLIN was hung over sleeping areas.

### Data analysis

Data analysis was done using Epi Info version 7.1.2.6. A child was considered to have utilised LLIN if he slept under an LLIN the night preceding the survey. Person-net ratio was computed by dividing the number of persons per household by the number of LLIN owned while the coverage was determined by the proportion of households with the ideal ratio of two persons to one LLIN (>0 - ≤2) Descriptive statistics was computed using frequencies, proportions and means. Bivariate analysis was done using odds ratios and 95% confidence interval was used to test for associations between utilization of LLIN and the factors. To identify the predictors of LLIN utilization, logistic regression analysis was conducted using factors with statistically significant associations at p < 0.25. For the logistic regression, significance level was set at p < 0.05.

### Ethical consideration

Prior to the commencement of the study, ethical approval was obtained from the Federal Capital Territory Health Research Ethics Committee Abuja (reference code: FHREC 2016/01/10 /22-02-16). Before inclusion in the study, informed consent was obtained from care givers of eligible children, and they were assured of confidentiality. They were also informed that non-participation in the study would not affect their stay or welfare within the camp in any way. To further ensure confidentiality, personal identifiers were removed from all data sets and access was password restricted.

## Results

**Figure 1:**
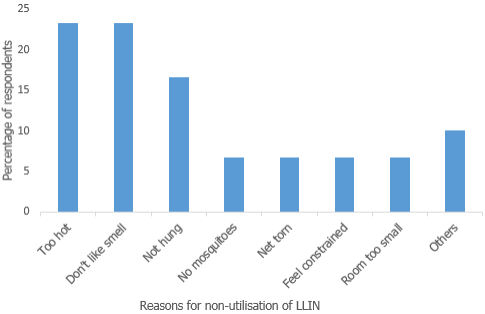
Reasons proffered for non-utilization of LLIN among children aged 6-59 months at IDP camps Abuja

A total of 393 children from 242 households were studied. Their mean age was 33.3 ± 17.4 months. About half of the participants were female 203 (51.6%) with the highest number of respondents from the Wassa IDP camp 233 (59.3%). The mothers were mainly in the 20-24 year age group 74 (32.5%), most had no formal education 104 (45.6%) and were Muslims 135 (57.5%) (Table 1). Of the households surveyed, 182 (75.8%) owned at least one LLIN while the coverage was 11.2% as only 26 households had the ideal person-net ratio. We also found that of those 290 children 6-59 months in households that owned LLIN, utilization was 89.7% as 260 children slept under an LLIN the night before the survey (Table 2). For the 30 children who didn’t sleep under the LLIN the night before the survey, the common reasons proffered was it "being too hot" 7 (25.0%) and "not liking the smell" 7(25.0%) and the “net not hung”5 (16.7%). Other reasons were "the room was too small", "yet to wash the LLIN" as instructed and had used "other mosquito preventive measures" (Figure 1).

**Table 1:** Demographic characteristics of children aged 6-59 months and their mothers at internally displaced persons camps Abuja

Characteristics	Frequency (N = 242)	Percentage (%)
**Age of children (months) (n = 393)**		
6-11	49	12.5
12-23	72	18.3
24-35	68	17.3
36-47	80	20.4
48-59	124	31.5
**Sex**		
Female	203	51.6
Male	190	48.4
**Camp site**		
New Kuchingoro	50	12.7
Durumi Area One	110	28.0
Wassa	233	59.3
**Age of Mother (years) (n=242)**		
15-19	5	2.2
20-24	74	32.5
25-29	73	32.0
30-34	39	17.1
35-39	24	10.5
>39	13	5.7
**Educational status of mother**		
No formal	104	45.6
Primary	40	17.5
Secondary	83	36.4
Tertiary	1	0.5
**Religion**		
Christianity	100	42.5
Islam	135	57.5
**Duration of stay in camp (months)**		
< 6	15	7.0
6-11	13	6.1
12-17	34	15.8
18-23	18	8.4
24-29	116	54.0
> 29	19	8.8

**Table 2:** Long-lasting insecticidal net ownership, coverage of households and utilization among children aged 6-59Months at internally displaced persons camps in Abuja

Variables	Frequency	Percentage(%)
Households with LLIN *(Ownership)[N =232]		
Yes	178	76.7
No	54	23.3
**HH members to LLIN ratio**+**(Coverage) [N =232]**		
> 0 - ≤ 2	26	11.2
> 2	152	65.5
0	54	23.3
**Children 6-59 months who slept under LLIN night prior to survey**-**(Utilisation) [N =290]**		
Yes	260	89.7
No	30	10.3

^+^Ownership: Availability of at least one LLIN in a household

+Coverage: Proportion of HH that have at least one LLIN to 2 persons per HH

-Utilisation: Child aged 6-59 months slept under an LLIN the night before the survey

The most common source of nets was NGO 107 (36.9%) and less than one third 88 (30.3%) of the nets had been purchased. Duration of LLIN ownership ranged from 2 days to 42 months with most of the participants 114 (39.9%) owning LLINs for 7 to 12 months. More than half of the LLINs, 158 (55.6%) had holes and 236 (81.7%) of the LLINs were observed hung at sleeping places during the time of the survey. The odds of using LLIN was higher among children in households where nets were observed hung in the home over sleeping area (odds ratio (OR) : 55.9, 95% confidence interval (CI) : 18.1-172.1), respondents that lived near bush and farmland (OR: 2.9, 95% CI: 1.33-6.59), had owned the LLIN for 6 months or less, (OR: 2.6, 95% CI: 1.2-5.6 ), were christians (OR:2.4, 95% CI: 1.1 -5.6) were also more likely to use LLINs. Additionally, respondents were more likely to use nets with holes (OR: 2.2, 95% CI:1.01-4.90), Other factors for net use which were not found to be statistically significant were child’s age and sex, mothers age and educational status and if nets were free or purchased. The independent predictors of LLIN utilization after controlling for other factors were LLIN observed hung over sleeping place (adjusted (aOR): 99.9, 95% CI: 22.7 - 438.7) and type of camp site (aOR: 8.2, 95% CI: 2.5-27.4) (Table 3).

**Table 3:** Factors associated with utilization of long-lasting insecticidal nets among children aged 6-59 months at internally displaced persons camps Abuja

Characteristics	Used LLIN	Did not use LLIN	OR	95% CI	p-value	AOR	95% CI	p-value
**Sex**								
Female	139 (90.3)	15(9.7)	1.1	0.54-2.45	0.78			
Male	121(89.0)	15 (11.0)						
**Age group (months)**								
<36	127 (90.7)	13 (9.3)	1.2	0.58-2.68	0.57			
36-59	133 (88.7)	17 (11.3)						
**Religion**								
Christianity	134 (93.7)	9 (6.29)	2.4	1.10-5.62	0.03	2.5	0.66-9.12	0.18
Islam	126 (85.7)	21 (14.3)						
**Mothers age (years)**								
≥36	32(88.9)	4(11.1)	1.7	0.60-5.26	0.21	2.6	0.51-13.52	0.25
≤35	216(81.8)	48(18.2)				1		
**Mothers educational status**								
Formal	119(79.3)	31(20.7)	0.6	0.36-1.20	0.09	0.6	0.13-1.70	0.31
No formal	128 (85.3)	22 (14.7)				1		
**Net seen hanging**								
Yes	232 (98.3)	4 (1.69)	55.9	18.12-172.14	< 0.001	99.9	22.7-438.8	<0.0001
No	27 (50.9)	26 (49.1)				1		
**Net purchased**								
Yes	77 (87.5)	11 (12.5)	0.7	0.33-1.60	0.43			
No	183 (90.5)	19 (9.41)						
**Duration of net ownership (months)**								
0-6	86 (83.5)	17 (16.5)	2.6	1.20-5.57	0.01	0.4	0.08-2.24	0.32
>6	170 (92.9)	13 (7.1)						
**Holes in LLIN**								
Yes	147 (93.0)	11 (7.0)	2.2	1.01-4.90	0.04	0.8	0.19-3.26	0.70
No	108 (85.7)	18 (14.3)						
**Breeding site type**								
Farmland/bush	205 (91.9)	18(8.1)	2.9	1.33-6.59	0.005	2.6	0.69-10.15	0.16
Stagnant water	46 (79.3)	12 (20.7)				1		
**Type of camp site**								
Camp-like site	103 (77.4)	30 (25.6)	0.6	0.37-1.16	0.08	8.2	2.5-27.4	0.0006
Settlement site	157 (83.9)	30 (16.1)				1		

## Discussion

This study investigated the coverage and utilization of LLIN among IDPs aged 6-59 months at IDP camps in Abuja, Nigeria. Overall, the study found high LLIN ownership in the households and utilization among children aged 6-59 months while the coverage was low. The independent predictors of utilization were LLIN observed hung over sleeping places and the type of camp site. This study showed high ownership of LLIN (76.7%) in households of IDPs aged 6-59 months. Even though the proportion is lower than that recommended by the WHO [2], it is higher than the LLIN ownership nationally and in the Federal Capital Territory Abuja 69% and 42% among children less than 59 months of age respectively [19]. The high ownership of LLIN at the IDP camps may be attributed to the fact that lots of the LLINs at the camp were donated through NGOs, national malaria elimination programme, religious organisations and were not purchased. Though ownership does not equate utilization [6], it is still an encouraging pointer towards achieving global targets in malaria elimination. The high ownership found in this study was similar to the finding among IDPs in Uganda where ownership was 75.6% [20]. This was also found after LLIN distribution campaign was done at the camps, so similar to our study, the LLIN were freely obtained. However, in the Democratic Republic of Congo, only 34% of households in the IDP camp had LLIN [6]. This may be attributed to the fact that unlike the finding of free LLIN distribution at the camps in the Abuja and Ugandan study, a low wealth quintile was established in the Democratic Republic of Congo camps. Thus, access to bed nets, if not freely distributed was limited as most households could not afford to purchase LLIN [6].

This study also found a low coverage rate of LLIN in the households. Coverage of LLIN is an important determinant of the effectiveness of this key strategy. This implies despite high ownership of LLIN, Children living in the IDP camps still fall short of accessing the protection afforded by LLIN. This low coverage found in our study may be a reflection of the low coverage rate found in the country in un-displaced populations as the NMIS found a coverage of 22.9% in Abuja [19]. Even though low, the rate is still twice the rate in IDPs aged 6-59 months of age. In a study among IDPs in Uganda, the coverage was appreciably higher than that found in our study( 56.9%) [20]. Though the coverage was assessed after mass distribution campaigns at the camp, there was no information as to whether distribution was done based on household size which could have accounted for the much higher coverage rate.

The strongest predictor of LLIN utilization in this study was net observed hanging at sleeping areas during the survey. The odds of using an LLIN was 100 times more among those who had their net hanging. This is understandable as it is more convenient to sleep underneath an already hung net than having to put up the net before sleeping. This corroborates findings in other studies [20,21]. Type of Camp site was another independent predictor of LLIN utilization determined in our study. Those IDPs aged 6-59 months living at camp like sites which consisted of tents made of tarpaulins in open fields, were eight times more likely to use LLIN than those living at abandoned housing shelters in settlement sites. It can be inferred that the houses though in state of disrepair offer better protection and housing than the tents which may influence the utilization of LLIN in both types of camps. With those exposed to poorer housing structures, more likely to utilise LLIN. Other variables not statistically significantly associated were age and educational status of the mothers, although IDPs aged 6-59 months whose mothers were 36 years of age and older were twice more likely to utilise LLIN than those with younger aged mothers. Our findings are subject to some limitations. utilization of LLIN, determined by history of LLIN use the previous night was not independently verified. Despite this, the study provides relevant information on the current situation of LLIN coverage and utilization in malaria prevention among displaced persons aged 6-59 months at IDP camps in Abuja, Nigeria. A special population in which there is dearth of information especially on malaria prevention. Another strength of this study is the sighting and observance of nets hanging over sleeping spaces which helped verify the responses proffered by care givers on LLIN utilization.

## Conclusion

Ownership and utilization of LLIN within the IDP camps in Abuja were high, emphasis should be on improving the low coverage rate which may affect effectiveness of LLIN in malaria prevention. Efforts to increase utilization of LIIN among the children should focus on improving household LLIN coverage rate by promoting further LLIN distribution and hanging campaigns.

### What is known about this topic

- Utilization of LLIN is protective against malaria;
- Utilization of LLIN is determined by its ownership and universal coverage;
- Utilization is low among vulnerable populations such as displaced persons.

### What this study adds

- Ownership and utilization of LLIN is high among IDPs in Abuja;
- Ownership of LLIN per household could be high while universal coverage remains low;
- Type of camp site is also an independent predictor of LLIN utilization.
